# Acceptability of multiple micronutrient powders and iron syrup in Bihar, India

**DOI:** 10.1111/mcn.12572

**Published:** 2017-12-06

**Authors:** Melissa F. Young, Amy Webb Girard, Rukshan Mehta, Sridhar Srikantiah, Lucas Gosdin, Purnima Menon, Usha Ramakrishnan, Reynaldo Martorell, Rasmi Avula

**Affiliations:** ^1^ Doctoral Program in Nutrition and Health Sciences, Laney Graduate School Emory University Atlanta Georgia USA; ^2^ Hubert Department of Global Health, Rollins School of Public Health Emory University Atlanta Georgia USA; ^3^ CARE India Patna Bihar India; ^4^ International Food Policy Research Institute Delhi India

**Keywords:** anaemia, India, iron and folic acid syrup, iron supplementation, multiple micronutrient powders, young children

## Abstract

Nearly two thirds of young children are anaemic in Bihar, India. Paediatric iron and folic acid syrup (IFAS) and multiple micronutrient powders (MNPs) are two evidence‐based interventions to prevent anaemia. Using a randomized crossover design, we examined the acceptability of IFAS versus MNPs for children 6–23 months. In a catchment area of 2 health centres in Bihar, health front‐line workers (FLWs) delivered either (a) IFAS twice weekly or (b) MNPs for 1 month followed by the other supplementation strategy for 1 month to the same families (NCT02610881). Household surveys were conducted at baseline (*N* = 100), 1 month after receiving the first intervention (1 month; *N* = 95), and 1 month after the second intervention (2 months; *N* = 93). Focus group discussions (10 FLWs) and in‐depth interviews (20 mothers) were held at 1 and 2 months. We used chi‐square and Fisher exact tests to test mothers' product preferences. Qualitative data were analysed using MaxQDA and Excel employing a thematic analysis approach. There was high adherence and acceptability for both products (>80%). There was no significant difference in preference (*p* < .05) on perceived benefits (39% MNPs, 40% IFAS), side effects (30% MNPs, 30% IFAS), ease of use (42% IFAS, 31% MNPs), child preference (45% IFAS, 37% MNPs), and maternal preference (44% IFAS, 34% MNPs). Mothers and FLWs indicated that the direct administration of IFAS ensured that children consumed the full dose, and MNPs intake depended on the quantity of food consumed, especially among younger children, which emphasizes the need to integrate supplementation with the promotion of optimal child feeding practices.

## 1 INTRODUCTION

Anaemia, a public health problem across the globe, affects 58% of children in South Asia (Stevens et al., 2013). In India, which contributes to a third of global burden of undernutrition, nearly 60% of children younger than 5 years old are anaemic. Bihar is one of the states in India with the highest burden of undernutrition. Despite modest declines over the past decade, anaemia persists in nearly two thirds of young children in Bihar (International Institute for Population Sciences and Macro International, 2017). This is of concern given the well‐established association of anaemia with impaired child growth and development and increased risk of morbidity and mortality (Black et al., 2013). Although there is growing global evidence on the diverse and complex aetiology of anaemia, it is estimated that approximately 42% of anaemia among young children in most low‐middle‐income countries is due to iron deficiency (Stevens et al., 2013).

Paediatric iron and folic acid syrup (IFAS) and multiple micronutrient powders (MNPs) are two evidence‐based strategies to prevent anaemia (Ministry of Health and Family Welfare, 2013; World Health Organization [WHO], 2016). The Government of India currently recommends IFAS to be directly administered via a 1‐ml dropper to all children (6–59 months) twice per week by front‐line workers called the Accredited Social Health Activists (ASHAs; Ministry of Health and Family Welfare, 2013). Important limitations of traditional oral iron supplementation include reported unpalatable metallic taste, teeth staining, potential for overdose, limited shelf life, poor compliance, perception of the syrup as medicine, lack of supplies, and procurement‐associated challenges (Mora, 2002; Nestel & Alnwick, 1997). Although a recent review has shown that direct administration of IFAS is an effective strategy for prevention and management of anaemia and improving compliance (Bairwa et al., 2017), additional information is needed to understand scalability in terms of the limitations of this intervention and burden on the health care system.

Home fortification with MNPs represents an innovative solution that addresses some of the challenges associated with the provision of paediatric iron syrup wherein the caregiver adds a daily dose of key micronutrients, including iron, directly to a child's meal within the home. MNPs are encapsulated with a thin vegetable‐based hydrogenated lipid coating that prevents changes in appearance, texture, or taste of the meal, making it more palatable to children (Zlotkin et al., 2004). MNPs have similar efficacy as IFAS with the added benefit of easy administration and high adherence (Zlotkin, Antwi, Schauer, & Yeung, 2003; Zlotkin, Arthur, Antwi, & Yeung, 2001). Administration of MNP does not require the supervision of a front‐line worker and can be provided in boxes with individually packaged sachets for daily household administration over several weeks. Currently, there is a strong global evidence base for MNPs and their impact on reducing anaemia (Bhutta et al., 2013; De‐Regil, Suchdev, Vist, Walleser, & Pena‐Rosas, 2011; WHO, 2016). According to a Cochrane review, home fortification with micronutrient powders reduced anaemia in young children by 31% and iron deficiency by 51% (De‐Regil et al., 2011). Given the strength of the evidence for the efficacy of this product, the WHO (2011, 2016) released strong recommendations in support of home fortification of foods with MNPs to improve iron status and reduce anaemia among young children. However, there are also challenges associated with the use of MNPs including the need for significant interpersonal behaviour change communication and maintenance of the supply chain (Strengthening Partnerships, Results, and Innovations in Nutrition Globally, 2015).

Given the high prevalence of anaemia among Bihar's children, there is a critical need to identify and evaluate multiple delivery strategies to help meet the daily nutrient requirements for child health and development. Although there is global evidence of the impact for both iron syrup and MNP in improving anaemia in young children, evidence is lacking regarding which strategy is most acceptable and likely to achieve coverage in India. To ensure the success of India's anaemia prevention and reduction programmes, it is critical that we identify the most acceptable approach both for families and for front‐line workers charged with delivering the supplement.

**Key messages**

- Both paediatric iron and folic acid syrup and multiple micronutrient powders are highly acceptable intervention options to prevent anaemia in rural Bihar, India.
- Implementation of either policy option will require consideration of front‐line worker workload, incentives, and delivery platform feasibility.
- There is a need to integrate supplementation programmes with the promotion of optimal child feeding practices, in areas with a high burden of anaemia.

## 2 METHODS

### 2.1 Study population

The study was a collaboration between CARE India, the International Food Policy Research Institute, and Emory University. We selected two health centre (HC) catchment areas in a single block of the West Champaran district in northern Bihar. Within each HC, we purposefully selected five ASHAs. From each ASHA's home visit roster, 10 households with a child 6–23 months of age were selected for a total of 50 households per HC (100 total households). Households were selected to maximize heterogeneity based on caste, child sex and age, sociodemographic characteristics, and distance from HC. The Indian Institute of Health Management Research, International Food Policy Research Institute's Institutional Review Board in India, and Emory University's Institutional Review Board approved the study. Informed consent was obtained from all study participants. Inclusion criteria were as follows: age between 6 and 23 months and not currently taking iron supplements. Exclusion criteria included wasting (mid‐upper arm circumference < 11.5 cm); suspected severe anaemia (through assessment of palmar pallor); history of haemoglobinopathy or repeated blood transfusions; and currently ill with pneumonia, fever, or acute diarrhoea. Children with wasting or suspected severe anaemia were referred to the nearest facility for care.

### 2.2 Study design

The study composed of a mixed‐method, longitudinal two‐arm, crossover design where 100 households with children 6–23 months of age were followed over the span of 2.5 months. HCs were randomized such that the ASHAs first delivered either (a) twice weekly IFAS or (b) MNPs for 1 month, which was then followed by the other supplementation strategy for 1 month with a 2‐week break in between. Thus, half of the households received IFAS followed by MNPs, and half received MNPs followed by IFAS (Figure S1). With this crossover design, each child had the opportunity to try both products and served as his or her own control. Analysis was conducted both stratified by HC and pooled. The trial was registered in ClincalTrials.gov (NCT02610881).

### 2.3 Intervention

ASHAs from our selected HCs were trained on the study protocol, product use, and infant and young child feeding (IYCF) practices before each round of product delivery. Each ASHA delivered either IFAS or MNP to 10 children within their catchment areas over the course of 1 month. Following a 2‐week washout period, ASHAs delivered the alternative product to the same children. While delivering the products, ASHAs counselled mothers on how to use the product and on IYCF practices and gave a pamphlet with information on IYCF (age‐appropriate complementary feeding messages on meal quantity, frequency, consistency, dietary diversity, and hand washing).

The ASHAs were asked to administer directly the IFAS using a 1‐ml dropper to each child in their catchment on Wednesday and Saturday over the span of 1 month for a total of eight visits from the ASHA (as per government policy). The iron syrup contained 20 mg elemental iron and 100 mcg folic acid per millilitre with caramel flavouring in a 100‐ml bottle (a separate bottle was used for each child).

In case of MNPs, ASHAs delivered 30 sachets (dose for 1 month) during their first visit and conducted a second follow‐up visit at 2 weeks to track product use and answer any questions pertaining to the product. Similarly, for IFAS, ASHAs counselled mothers on the product and gave an information pamphlet on IYCF and product use. The MNP product was administered by the caregiver and did not require the presence of the ASHA. The MNP contained 12.5 mg iron (ferrous fumarate), 5 mg zinc, 0.16 mg folic acid, 0.3 mg vitamin A, 30 mg vitamin C, 0.9 mcg vitamin B‐12, and 90 mcg iodine. The composition and packaging (local name *Jeevan Jyoti*, “light of life”) were produced specially for this context through earlier formative research (Young et al., 2014).

### 2.4 Data collection

We conducted household surveys at baseline (*N* = 100), after 1 month of use of Product 1 (1 month; *N* = 95) and after 1 month of use of Product 2 (2 months; *N* = 93). Surveys collected data on household demographics; child feeding practices; and product receipt, use, and acceptability. Acceptability was determined using a Likert scale (*agree*, *no opinion*, and *disagree*). Seven children were lost to follow‐up due to travelling for festivals or marriages. Focus group discussions were conducted with ASHAs (*N* = 5) at baseline, after 1 month, and after 2 months to understand their perceptions of the two products, ease of delivering them, workload, and incentives. In addition, we conducted semistructured interviews with 10 mothers per HC at the end of Month 1 and Month 2 to understand their perceptions of the two products, reasons for preference of one product over the other, and overall preference for the supplements.

### 2.5 Quality control

Extensive training was provided to enumerators prior to each data collection period. A team of nine enumerators collected quantitative data. Three field supervisors accompanied the data collection team to ensure quality and perform spot checks and back‐checks. Issues were immediately reported to the study coordinator. All surveys were edited prior to data entry, and a 10% back‐check was done on all surveys to ensure no errors were made during the entry process.

Qualitative data collection was conducted by three trained and experienced facilitators at baseline and four facilitators after 1 and 2 months. All qualitative data were translated and transcribed verbatim, and quality checks were conducted to ensure accuracy.

### 2.6 Statistical analyses

We summarize the descriptive data from surveys on household characteristics and experiences with the iron products using means/medians and frequencies and examine differences in caregiver product preferences using chi‐square and Fisher exact tests. All results were also stratified by HC and child age groups (6–15 months and 16–24 months). A *p*‐value less than .05 was considered significant. Polytomous logistic regression was conducted to model predictors of product preference. All quantitative data analyses were performed using SAS version 9.3 (SAS Institute, Cary, NC, USA). Qualitative data from semistructured interviews and focus group discussions were transcribed and translated. Interviews were analysed using MaxQDA and Excel employing a thematic analysis approach. Deductive themes defined a priori were coded and described, and inductive themes emerging from the data were also noted and coded.

## 3 RESULTS

A majority of households were Hindu (83%) and from deprived social strata belonging to ordinary backward caste (67%; Table 1). Product adherence was high for both IFAS (Table 2a) and MNP (Table 2b). In 96% of households, mothers reported children consuming IFAS twice a week and 83% reported receiving full 1 ml per time as per protocol. In over 80% of households, mothers likewise reported children consuming MNP as instructed (one sachet per day). Almost half of the mothers distributed the contents of one sachet across multiple meals per day, a strategy recommended by some ASHAs if families reported that children were eating only a small amount of food or in cases of upset stomach. There were no differences in product adherence by HCs.

**Table 1:** Sample characteristics of children and households^a^

Characteristics	Health Centre 1 (%)	Health Centre 2 (%)
Gender		
Female	50	60
Age (months)		
6–12	32	52
13–18	48	36
19–23	20	12
Religion		
Hindu	92	74
Muslim	8	24
Caste		
Scheduled caste or tribe	34	22
Other backward class	56	78
Other	10	0

a Health Centre 1 was randomized to first iron and folic acid syrup (IFAS) then multiple micronutrient powders (MNPs), *n* = 50; Health Centre 2 was randomized to first MNP then IFAS, *n* = 50.

**Table 2a:** Adherence to iron and folic acid syrup (IFAS) by health centre (HC)^a^

	HC 1 (%)	HC 2 (%)
Frequency of product consumption		
>1 per day	0	2
Once per day	0	2
Twice a week	98	93
Once a week	2	0
Few times a month	0	2
Amount of product given per day		
<1 dropper	16	18
1 dropper	84	82
>1 dropper	0	2
Child took product as instructed		
Yes, always or most of time	90	89
Yes, about half the time	4	0
No, usually not	6	7
No response	0	4

a HC 1 was randomized to first IFAS then multiple micronutrient powders (MNP), *n* = 50; HC 2 was randomized to first MNP then IFAS, *n* = 50.

**Table 2b:** Adherence to multiple micronutrient powders (MNPs) by health centre (HC)^a^

	HC 1 (%)	HC 2 (%)
Frequency of product consumption		
More than once per day	42	46
Once per day	58	54
Amount of product given per day		
<1 sachet	18	4
1 sachet	80	96
>1 sachet	2	0
Child took product as instructed		
Yes, always or most of time	83	85
Yes, about half the time	7	0
No, usually not	11	8
No response	1	2

a HC 1 was randomized to first iron and folic acid syrup (IFAS) then MNPs, *n* = 50; HC 2 was randomized to first MNP then IFAS, *n* = 50.

MNP and IFAS product acceptability was assessed after each round of exposure to the products at the end of month of product trial period per product (Table 3). The majority of mothers reported that their children consumed the iron product in the past month and expressed interest in continuing to use the product in the future (91–100%). Mothers perceived that both products provided positive benefits to their children, which included increased energy (82–88%), increased food consumption (78–88%), increased mental activity (82–88%), and improved child health (74–90%). Over 90% of mothers acknowledged the importance of giving IFAS and MNP to their child. One mother who used MNP said, “… so many benefits have occurred …. She became more fat,” and another mother who used IFAS said, “Cold and fever got over after taking medicine.”

**Table 3:** Caregiver reported multiple micronutrient powders (MNPs) and iron and folic acid syrup (IFAS) product acceptability by health centre (HC)^a^

	IFAS		MNP	
Product questions: (%, yes)	HC 1 (%)	HC 2 (%)	HC 1 (%)	HC 2 (%)
Child ever consumed product	98	98	100	96
Would like to continue using the product	100	93	94	98
Reported increased energy	86	82	87.5	82
Reported increased food consumption	88	78	85	82
Reported increased mental activity for child	88	82	87.5	82
Reported improved child's health	86	89	90	74
Important to give product to child	98	91	96	98
I think my child liked this product	98	95.5	94	87
I think it was easy to give product to my child	100	98	96	100
I want to continue using this product	100	91	94	98
Would you be interested in purchasing	98	78	77	91

a HC 1 was randomized to first IFAS then MNPs, *n* = 50; HC 2 was randomized to first MNP then IFAS, *n* = 50.

MNP and IFAS product preference was assessed after mothers had the opportunity to try both products (Table 4). The order in which mothers received the products influenced product preference, with higher preference for the last product offered. Thus, in HC 1, which was randomized to receive IFAS first and MNP second, the majority of the mothers (60%) preferred MNP, whereas in HC 2, the majority of mothers (56%) preferred IFAS. In addition, the results of the polytomous logistic regression model of product preference (MNP, IFAS, both, or neither) confirmed that the only significant predictor of product preference was order of product receipt after controlling for age, sex, caste, and religion (data not shown). Overall, there was no significant difference in the preference for either product based on ease of use (42% IFAS, 31% MNPs, 25% both, and 2% neither), side effects (30% IFAS, 30% MNPs, 2% both, and 38% neither), child preference (45% IFAS, 37% MNPs, 16% both, and 2% neither), or caregiver preference (44% IFAS, 34% MNPs, 19% both, and 2% neither). Mothers expressed nearly equal preference to continued using of IFAS (38%) and MNPs (38%), with only one mother not wanting either product and 24% mothers preferring both products. Product preference did not significantly vary by child age group.

**Table 4:** Caregiver reported multiple micronutrient powders (MNPs) and iron and folic acid syrup (IFAS) product preference by health centre^a^^,^ b

	Which was easier to use? (%)	Which had fewer side effects? (%)	Which has the greatest benefits to the child? (%)	Which do you think the child preferred? (%)	Which did you prefer? (caregiver; %)	Which would you prefer to continue? (%)
Health Centre 1						
MNP	50	46	58	58	57	60
IFAS	21	21	23	25	21	21
Both	27	0	17	15	21	17
Neither	2	33	2	2	2	2
Health Centre 2						
MNP	11	13	18	13	16	11
IFAS	64	40	60	67	69	56
Both	22	4	20.5	18	18	31
Neither	2	42	2	2	2	2
Overall						
MNP	31	30	39	37	34	37
IFAS	42	30	40	45	44	38
Both	25	2	18.5	16	19	24
Neither	2	38	2	2	2	2

a Health Centre 1 was randomized to first IFAS then MNPs, *n* = 50; Health Centre 2 was randomized to first MNP then IFAS, *n* = 50.

b Preference data obtained at 2 months from 93 households after households had tried both products; overall, there were no statistical differences in product preference (*p* > .05).

The role of child feeding practices and necessity to provide MNP with a child's meal had both a positive and negative influence on maternal product preference. A mother who used MNP in the second round said, “Powder is preferable because the child who may not be eating rice will also start eating …. I prefer feeding baby myself.” Another mother who used MNP in the first round said, “If powder is there, sometimes they will eat or sometimes they will throw/scatter …. It is to be fed with rice ... but if it is from the bottle [referring to IFA], it can be directly fed, so child will neither throw nor litter.”

Mothers in both HCs noted that MNP was better suited for older children who had “become smart” and were able to eat on their own. The ASHAs expressed a similar opinion: “When we give powder for children aged 2–3 years it is easy and they will eat happily, but not babies. Babies and smaller children do not eat food properly and there are lots of problems. Mothers complain that even though they mix the powder with food, sometimes their child does not eat all the food and leaves something behind.”

Both ASHAs and mothers stated that using the iron syrup was easier and allowed assurance that the child was receiving the full dose of the supplement, as compared with MNP where the amount consumed depended on the quantity of food the child ate. As illustrated by a mother who commented that she liked the syrup as her child, “was not wasting … use to drink (the iron syrup) easily.” Some mothers and ASHAs perceived that fidelity to iron syrup was higher because the ASHAs ensured the child was consuming the product twice weekly; administration by the ASHAs was believed to reduce the burden on mothers as well. One ASHA commented that both products were equally good, “For us (ASHAs) both (IFAS and MNPs) are fine … now it depends on the caregiver because we do not know whether she is feeding or not, but iron syrup we are feeding on our own … so we prefer it.”

ASHAs were particularly happy with the iron syrup product citing that children were responding very well to it and that it was commonly referred to as a “medicine.” ASHAs reported, “Mothers say this [IFAS] is very beneficial medicine, can you bring two more and give me? Everyone is praising this medicine.”

ASHAs reported that the distribution of the iron‐containing products enhanced their relationship with the community but had challenges with distributing them only for a few selected children. ASHAs reported that both other children and mothers themselves requested the iron products: “they [mothers] see that we are feeding other children and not feeding their child … so we have become bad in their eyes.”

Although ASHAs were willing to distribute either product, they expressed an overall preference for the iron syrup in the qualitative interviews that related to the ease of use: “Syrup can be given easily in one time only, so they will also feel that child is having something, while the powder has to be given several times and they forget to give it, but syrup can be given in one time.”

When asked about scale up, an ASHA commented that the delivery of the supplement may depend on households: “if they understand we will give them one dose and then hand it over to the beneficiary”; in other homes, more direct support might be needed.

ASHAs in one HC attributed seasonality to maternal preference of IFAS to MNPs: “Jeevan Jyoti (MNP) was distributed in winter. So if anyone fell ill then they felt it may be due to Jeevan Jyoti … while we gave syrup it was spring, it was neither too hot nor too cold, and it has suited children as well … so we came to understand that this syrup is good.”

The role of season was not mentioned in the other HC.

When asked how the product should be distributed to communities in the future, one ASHA responded, “If ASHA is given incentive per day then it should be done through ASHA only.” As ASHAs are not salaried workers and are paid based on the services they deliver, ASHAs expressed a need for incentives to justify taking on a new task of delivering either IFAS or MNPs: “We are satisfied but unhappy because of salary, which we have not received. We feel demotivated. We do a lot of work and every department is proud of us, that our ASHAs are field workers but the government not giving salary demotivates us.”

ASHAs were then asked to state for which product they should require greater incentives. Although some answered, “We should get more incentives in both,” others expressed that higher incentives would be needed with IFAS distribution compared with MNP due to increased work load (eight visits per month with IFAS; two visits per month with MNP).

## 4 DISCUSSION

We found overall high adherence and acceptability for both IFAS and MNPs among mothers and ASHAs in Bihar, India, as supplements for 6‐ to 23‐month‐old children. This research aligns with a recent systematic review of MNP use that reported high acceptability and good adherence (50–90%; de Barros & Cardoso, 2016). The higher rates of adherence in our study (>80% reported taking the MNP and IFAS as instructed) may have been due to the short duration of follow‐up and/or more intensive counselling.

One of the key strengths of our study is the randomized crossover design where both products were provided to the same mother–child pair. This study design reduces confounding factors at the mother and child level. At the end of the study, mothers were evenly split on preference for which product to continue, and some ASHAs preferred IFAS due to perceived lower “waste” of product. Few studies have carefully compared different delivery options. We found one study that was conducted in a low‐income U.S. population, and although adherence was poor for both MNPs (31–46%) and iron drops (32–63%), similar to our study, there was no significant difference in which product the families would use again (Geltman et al., 2009).

IFAS and MNP also had similar perceived benefits and side effects. Overall, mothers reported numerous benefits such as increased energy, greater food consumption, and improved child health and development for both products while reporting only minor side effects for either product. This aligns with prior reports on household acceptability and side effects such as diarrhoea, vomiting, and constipation (de Barros & Cardoso, 2016).

Two factors that appeared to influence preference were (a) the order in which families received the product (with families reporting to prefer whichever product they had last) and (b) child's age. Prior research has also reported an influence of product order in acceptability trials. Novelty of products and recall bias can influence the way respondents evaluate the first versus the second product tried (Dibari et al., 2013; Gunaratna, Bosha, Belayneh, Fekadu, & De Groote, 2016; Schlich, 1993), thus, emphasizing the need for a randomized crossover design to evaluate product acceptability. Although product preference by child's age did not vary significantly in our quantitative data, the findings from the qualitative research suggested a slight preference for iron syrup for younger children who would “waste less”. MNP was perceived as better suited for older children who had “become smart” and were able to eat on their own.

Delayed introduction of complementary foods is a major concern in Bihar with less than a third of children 6–8 months receiving any solid or semi‐solid food and only 7.5% of children 6–23 receiving a minimum adequate diet (International Institute for Population Sciences and Macro International, 2017). Messages on product use need to be integrated with age‐appropriate IYCF messages and counselling. Prior studies have reported the need for MNPs to be mixed with food and increased appetite as potential barriers to MNP use in food‐insecure areas (de Barros & Cardoso, 2016; Tripp et al., 2011). However, despite household food insecurity, mothers in this study and in other studies viewed the increased appetite of the child and weight gain positively (Tripp et al., 2011). Furthermore, MNPs' food requirement is another opportunity to improve children's health beyond supplementation (Cardoso et al., 2016). Additional research is required to understand better the implications of MNPs versus iron and folic acid supplementation on child feeding practices.

ASHAs reported high acceptability and feasibility of delivering either the IFAS or MNP. Incentives for ASHAs will need to be appropriate for level of work involved. In this study, some ASHAs reported a preference for IFAS because they could ensure that the child received the full dose, and the increased visits (eight visits per month with IFAS vs. two visits per month with MNP for each child) may provide opportunities for further incentives. Currently, plans for incentivizing ASHAs to implement the existing IFAS programme are unclear as is how this may change if MNPs were adopted.

Both the crossover study design and use of mixed methods are important strengths of our study. The crossover design allowed us to take into account the effects of recall bias on product preference; for example, if we had given all children IFAS followed by MNP, we may have arrived at false conclusions on product preference and vice versa. However, the short study duration, novelty of both iron products, and the high ASHA–child ratio may have motivated the mothers to view the products favourably. Another important limitation of our study is sample size. Due to feasibility issues, ASHAs were asked to each visit only 10 children per month to allow us to collect detailed information on household and community front‐line worker perspectives and preferences. However, in practice, ASHAs serve a much larger population (typically one ASHA per catchment area of 1,000 residents, including roughly 100 children). Conducting eight visits per month to administer IFAS for each eligible child would be logistically challenging. This could lead to poor coverage, which is not ideal for an acceptability study. As a result, the total work burden of the two approaches could not be fully examined in this study and would require examination of a wider scale implementation of the programme. For the purposes of this study, we used the existing Indian guidelines for IFAS supplementation and the WHO guidelines for MNP (Ministry of Health and Family Welfare, 2013; WHO, 2016). Future research on anaemia reduction strategies would benefit from knowledge of the aetiology of anaemia, exploring alternative dosing and delivery platforms for scalability and sustainability. In particular, additional research of the feasibility of having the iron syrup delivered directly by caregivers should be examined.

In conclusion, both iron products are acceptable policy options for preventing paediatric anaemia. This work emphasizes the need to integrate supplementation with the promotion of optimal IYCF practices. Given the high prevalence of suboptimal feeding practices in Bihar, any iron supplementation strategy, whether IFAS or MNPs, should be supported with strong implementation of age‐appropriate counselling on appropriate complementary feeding practices to maximize impacts on child health.

## CONFLICTS OF INTEREST

The authors declare that they have no conflicts of interest.

## CONTRIBUTIONS

MY, AWG, RM (Mehta), SS, PM, UR, RM (Martorell), and RA designed the research; RM (Mehta) conducted the field research; MY, RM (Mehta), LG, AWG, and RA analysed data; MY, AWG, RM (Mehta), and RA wrote the manuscript. MY had primary responsibility for the final content of the manuscript. All authors read and approved the final manuscript.

## Supporting information

Figure A1. Participant flow chart
