# Supplementary figures and images for: Acceptability of multiple micronutrient powders and iron syrup in Bihar, India
Source: Matern Child Nutr. 2017 Dec 6;14(2):e12572. doi: 10.1111/mcn.12572 (PMC5900720; doi:10.1111/mcn.12572)

**SUPPLEMENTARY APPENDIX**

**Figure 1: Participant flow chart**


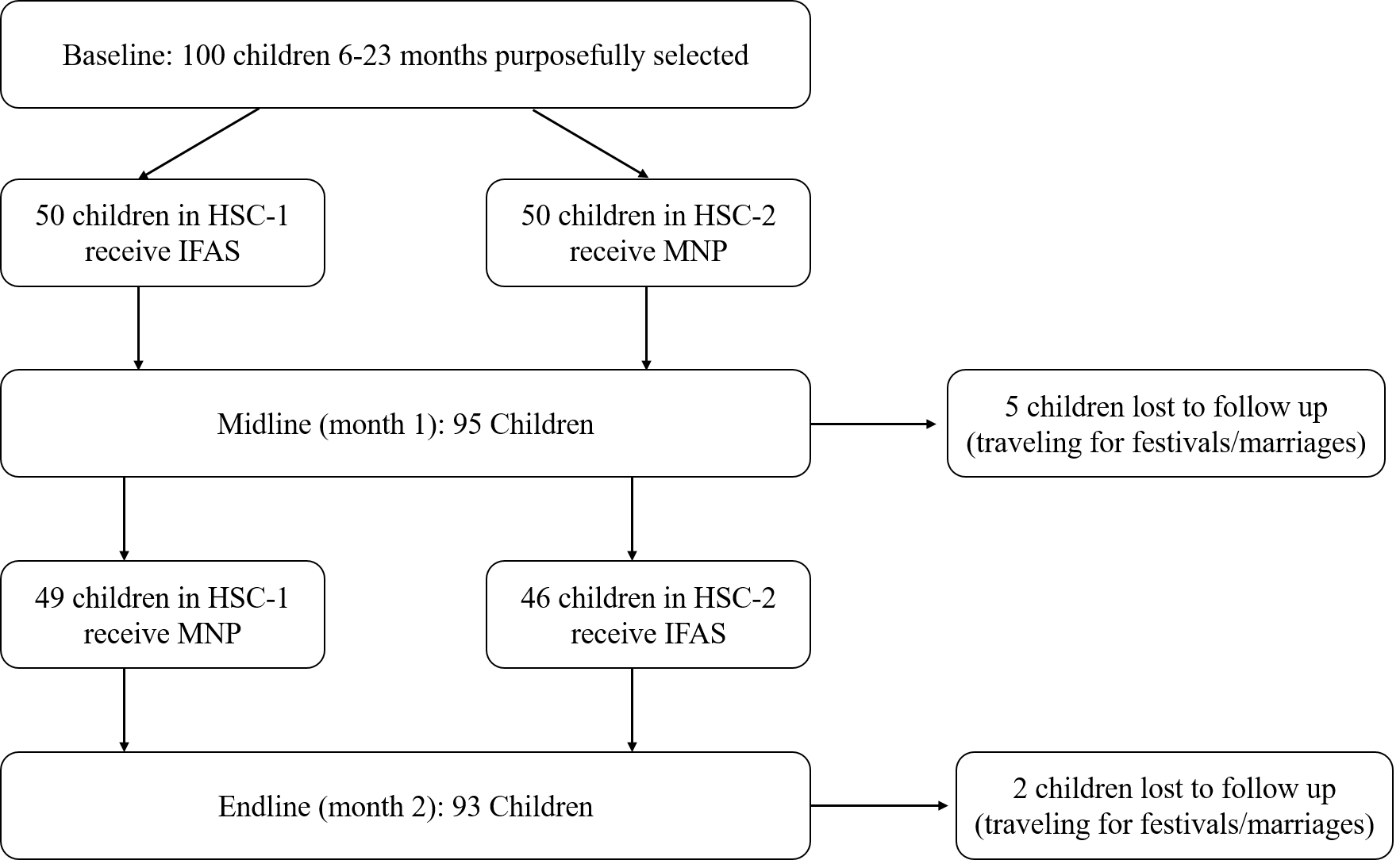

Supplement: Supplementary file 1 — Figure A1. Participant flow chart [file MCN-14-e12572-s001.docx]
